# Histopathological features of glandular atrophy of the lamina propria of the gastric mucosa during its occurrence and development

**DOI:** 10.1186/s12876-023-03033-6

**Published:** 2023-11-15

**Authors:** Yang-kun Wang, Dong-mei Ran, Ying-ying Li, Chao-ya Zhu, Ren-bing Zhang, Bo Jiang, Su-nan Wang

**Affiliations:** 1 Department of Pathology, The Fourth People’s Hospital of Longgang District, Shenzhen, 518123 China; 2 Department of Pathology, Southern University of Science and Technology Hospital, Shenzhen, 518055 China; 3 Shenzhen Polytechnic, Xili Lake, Xilihu Town, Nanshan District, Shenzhen, Guangdong 518055 China; 4 Third Affiliated Hospital of Zhengzhou University, Shenzhen, 450052 China; 5 Department of Pathology, Shenzhen Longgang District People’s Hospital, Shenzhen, 518172 China; 6 Department of Pathology, No. 990 Hospital of the PLA Joint Logistics Support Force, Zhumadian, 463000 China

**Keywords:** Immunohistochemistry, Intraepithelial neoplasia, Gastric adenocarcinoma, Gastric mucosal atrophy, Histopathology

## Abstract

**Objective:**

To explore the histopathological features of glandular atrophy of the lamina propria of gastric mucosa during its occurrence and development.

**Method:**

We performed detailed histological observation and immunohistochemical examination on the endoscopic biopsy and ESD endoscopic resection specimens of 896 patients with glandular atrophy of the lamina propria of gastric mucosa. The EnVision two-step method was used for immunohistochemical staining, and the slices were incubated with primary antibody CK7, CK20, villin, CDX2, MUC5AC, MUC6, p53 and ki-67. Hematoxylin staining was performed and observed under the microscope and statistically analyzed.

**Results:**

In the initial stage of glandular atrophy of the lamina propria, the proliferation area of the deep gastric pits, and the isthmus and neck of the gastric glands are characterized by roughly normal structure of the glandular structure, increased mesenchyme, and widened space between glands. Subsequently, the gland becomes smaller in volume and less in number, especially at the base, in the gastric glandular part of the gastric unit. The disease at this stage has higher incidence, and occurs more often in the elderly who account for 64.0% (573/896) of our study group. The disease in this stage may exhibit some lesions that are physiologic (age-related degeneration) while others are pathological. Therefore, this condition is called simple glandular atrophy of the lamina propria of the gastric mucosa. When the gastric mucosal epithelium is subjected to infection or repeated infections, chemical stimuli, immune factors, and genetic factors, it can lead to the proliferation and transformation of stem cells in the proliferation area of the deep gastric pits, and the isthmus and neck of the gastric glands, forming single ducts, multiple ducts, or a proliferation of patchy cells. Then, atypical hyperplasia (intraepithelial neoplasia) presents, finally leading to gastric adenocarcinoma.

**Conclusion:**

Understanding the histopathological characteristics of glandular atrophy of the lamina propria of gastric mucosa is of great significance in controlling the occurrence and development of gastric cancer.

## Introduction

In recent years, with the use of a variety of novel gastroscopes such as virtual endoscopy, magnifying endoscopy, and ultrasound endoscopy, the standardization of histopathological diagnosis in endoscopic biopsy has become important [1–3]. Gastric mucosal atrophy is an important diagnosis of gastric mucosal biopsy tissue, and is one of the most common lesions found clinically worldwide [4–6]. It has been reported that atrophic and non-atrophic lesions in gastric mucosa can be distinguished by endoscopic observation of changes in the color of the gastric mucosa, and the boundaries of mucosal atrophy can be identified [7, 8]. In histopathology, the classification and grading of gastritis and the distinction between atrophy and non-atrophy are emphasized [9]. Most gastric cancers develop in the context of atrophic gastritis and the risk increases with the degree of atrophy [10]. There is still considerable disagreement among pathologists on the definition and severity degree of gastric atrophy. In particular, gastric mucosal atrophy has not been completely differentiated from atrophic gastritis. Moreover, chronic atrophic gastritis is classified into 3 grades, namely mild, moderate, and severe. Mild: the number of lamina propria glands decreases by 1/3rd of the original ones; Moderate: the number of lamina propria glands decreases by 1/3rd − 2/3rd of the original ones; Severe: the number of lamina propria glands decreases by more than 2/3rd of the original ones. It is worth noting that the current international recommendation is to use OLGA/OLGIM to assess the severity of gastric mucosa atrophy, and can stratify the risk of gastric cancer in patients, which is conducive to the monitoring and follow-up of atrophy. Among them, stage III and IV in the OLGA/OLGIM system are high-risk patients for gastric cancer [4, 11–14]. In the compensatory proliferative atrophy period of gastric mucosal atrophy, the thickness of the gastric mucosa does not decrease but increases, hence the thickness of the mucosa cannot be used as diagnostic criteria for gastric atrophy.

Our previous studies and follow-up of gastric mucosal atrophy have found that the occurrence of gastric mucosal atrophy lesions is mainly caused by *H.pylori* infection. For controlling the occurrence and development of gastric mucosal atrophic lesions, it is crucial to control or eradicate *H.pylori* infection and prevent further growth into stem cell proliferation disorders in the proliferation region [15, 16]. In this study, we collected 896 cases of glandular atrophy of the lamina propria of gastric mucosa, and further studied the occurrence and development of gastric mucosal glandular atrophy, which finally led to identifying the histopathological characteristics of gastric adenocarcinoma and its precancerous lesions at various stages. We aim to provide accurate basis for clinicians to treat gastric mucosal atrophic lesions, track the transformation of malignant cells, as well as better control the occurrence and development of gastric cancer.

## Materials and methods

### Materials

A total of 896 patients with glandular atrophy of the lamina propria of gastric mucosa, diagnosed by histology of gastroscopic biopsy between November 2019 and June 2020 in the Southern University of Science and Technology Hospital, the Third Affiliated Hospital of Zhengzhou University, the Shenzhen Longgang District People’s Hospital, and the 990th Hospital of the Joint Logistics Support Force of the People’s Liberation Army, were enrolled in this study. Among them, 32 cases of glandular atrophy high-grade intraepithelial neoplasia and 11 cases of intramucosal tubular papillary adenocarcinoma were subjected to endoscopic submucosal dissection (ESD). The remaining 853 cases with glandular atrophy of the lamina propria of gastric mucosa underwent biopsy. There were 31 cases of gastric angle biopsy, 24 cases of gastric antrum biopsy, 17 cases of gastric body biopsy, and 11 cases of gastric fundus biopsy. A total of 3–5 pieces of mucosal tissue were obtained from each biopsy area. Specimens were fixed with 10% neutral formalin, routinely dehydrated, paraffin-embedded, and sectioned into 4 μm slices for HE and immunohistochemical staining.

### Immunohistochemical staining

The EnVision two-step method was used for staining. First, the paraffin wax in the tissue sections was removed, hydrated, and rinsed with distilled water. Then, the sections were placed in TBS for 10 min, blocking endogenous peroxidase for 5 min, followed by treatment of sections with TBS for 10 min. The slices were incubated with each primary antibody (CK7, CK20, villin, CDX2, MUC5AC, MUC6, p53, ki-67) for 30 min at room temperature. After washing in TBS for 10 min, the slices were incubated in EnVision™. Then the slices were washed with TBS for 10 min, then the secondary antibody was applied for 10 min. Then, the slices were incubated with chromogenic substrate solution for 10 min and rinsed with distilled water. Color was developed using DAB staining, and the slices were counterstained with hematoxylin. The known gastric mucosal sections were used as positive controls, while PBS buffer was used as a negative control instead of primary antibodies. The working solutions were all purchased from Fuzhou Maixin Biotechnology Development Co., Ltd., and the procedure steps were carried out in strict accordance with the instructions on the kit.

### Statistical analysis

The SPSS 22.0 software was used for statistical analysis. Counting data were expressed in terms of frequency and percentage. The age was divided into ≤ 60 years old and > 60 years old, and the degree of adenoid atrophy of lamina propria of gastric mucosa was compared between different ages and different genders. Chi-square test was used to compare the differences in pathological status between different levels. A P value of less than 0.05 was considered statistically significant.

## Results

### Association between age of onset and gender in glandular atrophy of the lamina propria of gastric mucosa

There were 545 males and 351 females diagnosed with glandular atrophy of lamina propria, with high proportion of males than females in each stage. There were 55.0% patients who had an age of onset of glandular atrophy of lamina propria ≤ 60 years old (493/896), while 45.0% patients were aged > 60 years (403/896) at onset. There were more patients aged ≤ 60 years at onset than those aged > 60 years at onset, as shown in Table 1.

### Clinical characteristics

**Fig. 1:**
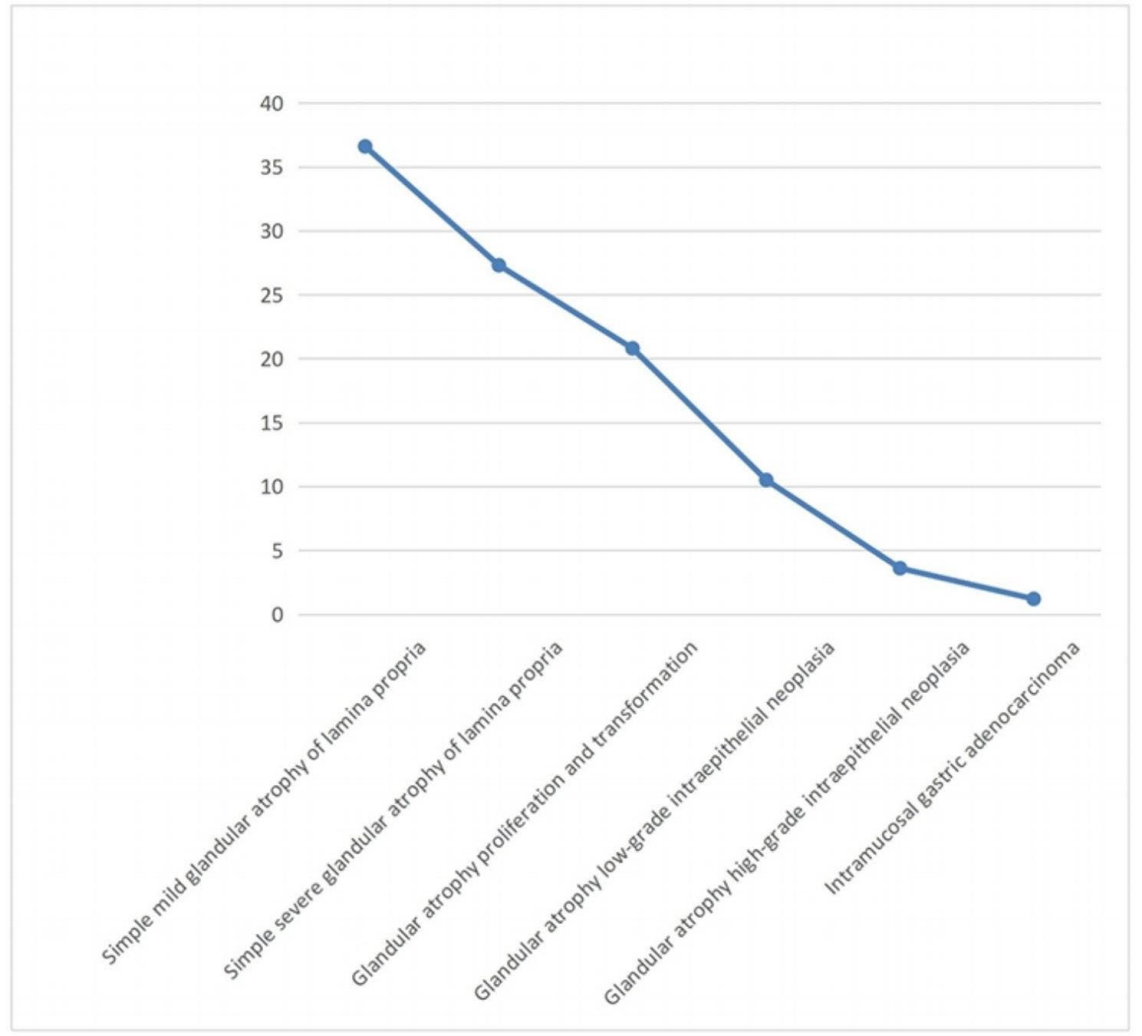
Development and changes in glandular atrophy of the lamina propria of gastric mucosa (n = 896)

Of these cases, simple mild atrophy of lamina propria glands accounted for 36.6% (328/896), 27.3% (245/896) had simple severe atrophy of lamina propria glands, 20.8% (186/896) had glandular atrophy proliferation and transformation, 10.5% (94/896) had glandular atrophy low-grade intraepithelial neoplasia, 3.6% (32/896) had glandular atrophy high-grade intraepithelial neoplasia, and 1.2% (11/896) had gastric adenocarcinoma, as shown in Fig. 1.

**Table 1:** Association between age of onset and gender in glandular atrophy of the lamina propria of gastric mucosa (n = 896)

Stage	N(%)	Gender	Age
		Male/Female	≤ 60(%),>60(%)
Simple mild glandular atrophy of lamina propria	328(36.6)	203/125	197(60.1),131(39.9)
Simple severe glandular atrophy of lamina propria	245(27.3)	148/97	142(57.9),103(43.9)
Glandular atrophy proliferation and transformation	186(20.8)	109/77	89(47.8),97(52.2)
Glandular atrophy low-grade intraepithelial neoplasia	94(10.5)	58/36	46(48.9),48(51.1)
Glandular atrophy high-grade intraepithelial neoplasia	32(3.6)	20/12	14(43.8),18(56.3)
Intramucosal gastric adenocarcinoma	11(1.2)	7/4	5(45.5),6(54.5)

### Histopathological features of glandular atrophy of the lamina propria of gastric mucosa

**Fig. 2:**
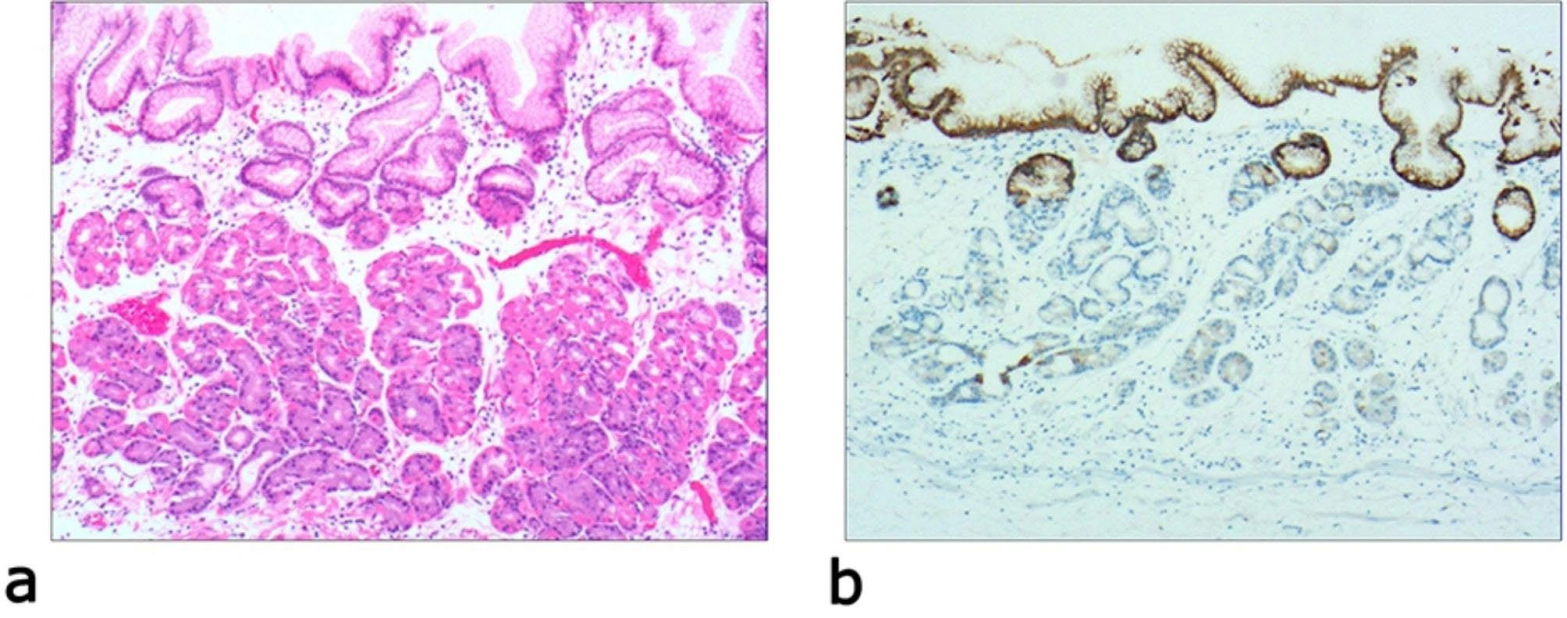
Simple mild glandular atrophy of lamina propria, **a**: glandular presented with roughly normal structure in the morphology in the proliferative area of the deep gastric pits, and the isthmus and neck of the gastric glands, which presents with increased mesenchyme and widened glands. The number of basal gastric glands is reduced by 1/2 of the original ones. H&E staining (× 40). **b**: The number of MUC5AC-positive cells is reduced. EnVision method (× 40)

**Fig. 3:**
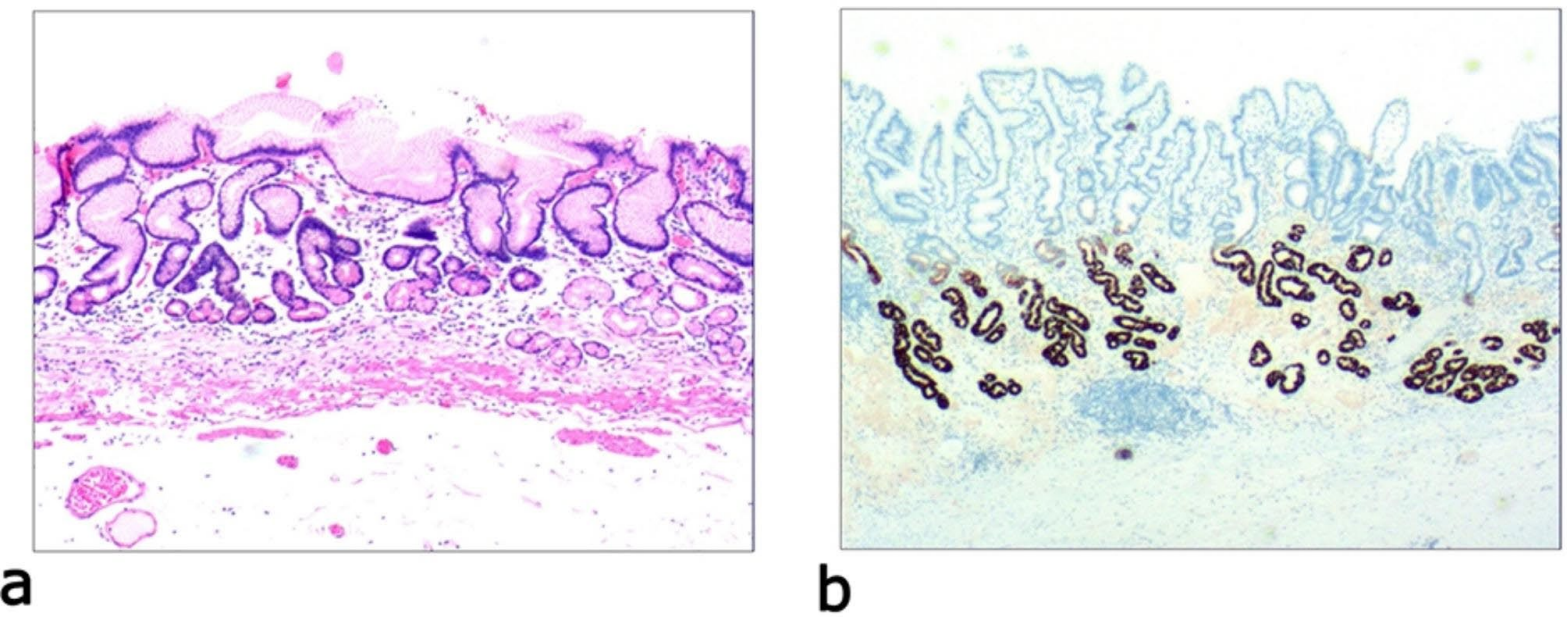
Simple severe glandular atrophy of lamina propria, **a**: glandular presented with roughly normal structure in the morphology in the proliferative area of the deep gastric pits, and the isthmus and neck of the gastric glands, which presents with more obviously increased mesenchyme and widened glands. The number of basal gastric glands is reduced by more than 1/2 of the original ones. H&E staining (× 40). **b**: The number of MUC6-positive cells is reduced. EnVision method (× 40)

**Fig. 4:**
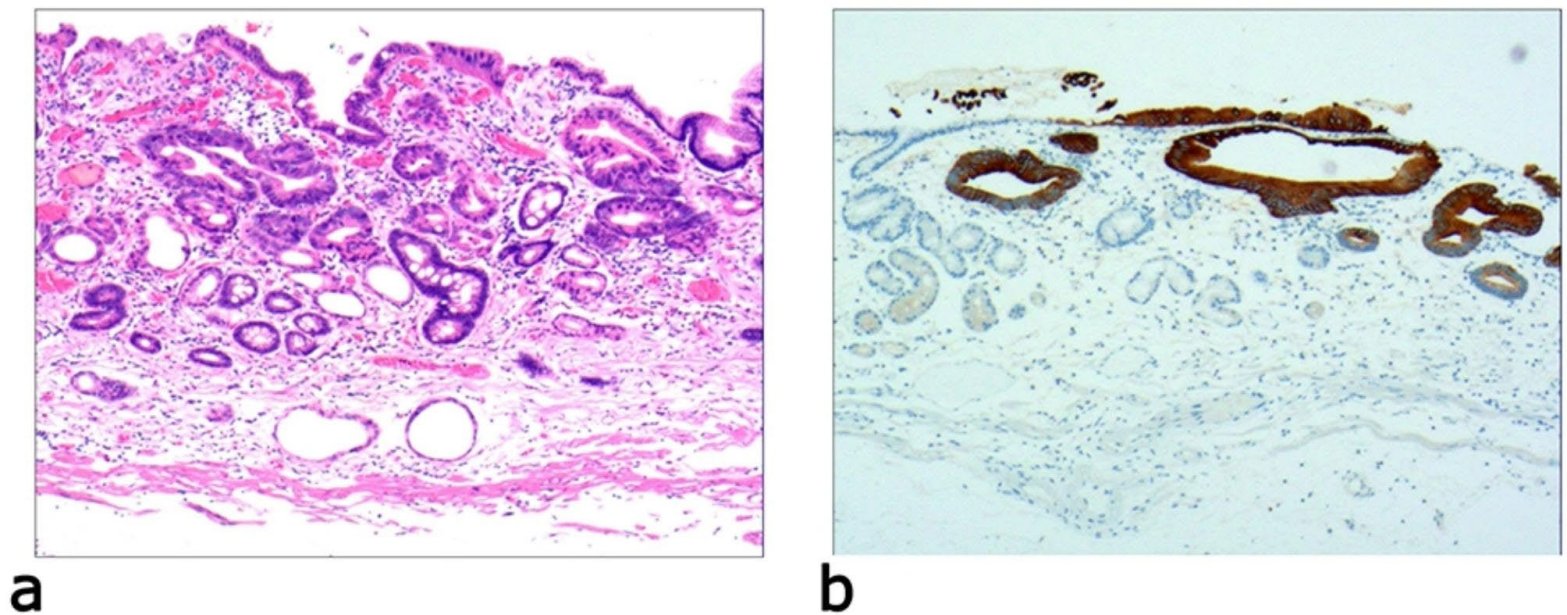
Glandular atrophy proliferation and transformation, **a**: significant proliferation in the proliferation area or proliferation band area of the deep gastric pits, and the isthmus and neck of the gastric glands. Formation of single ducts or multiple ducts, or proliferation of patchy cells. The hyperplastic glands are slightly expanded. H&E staining (× 40). **b**: positive expression of CK20. EnVision method (× 40)

**Fig. 5:**
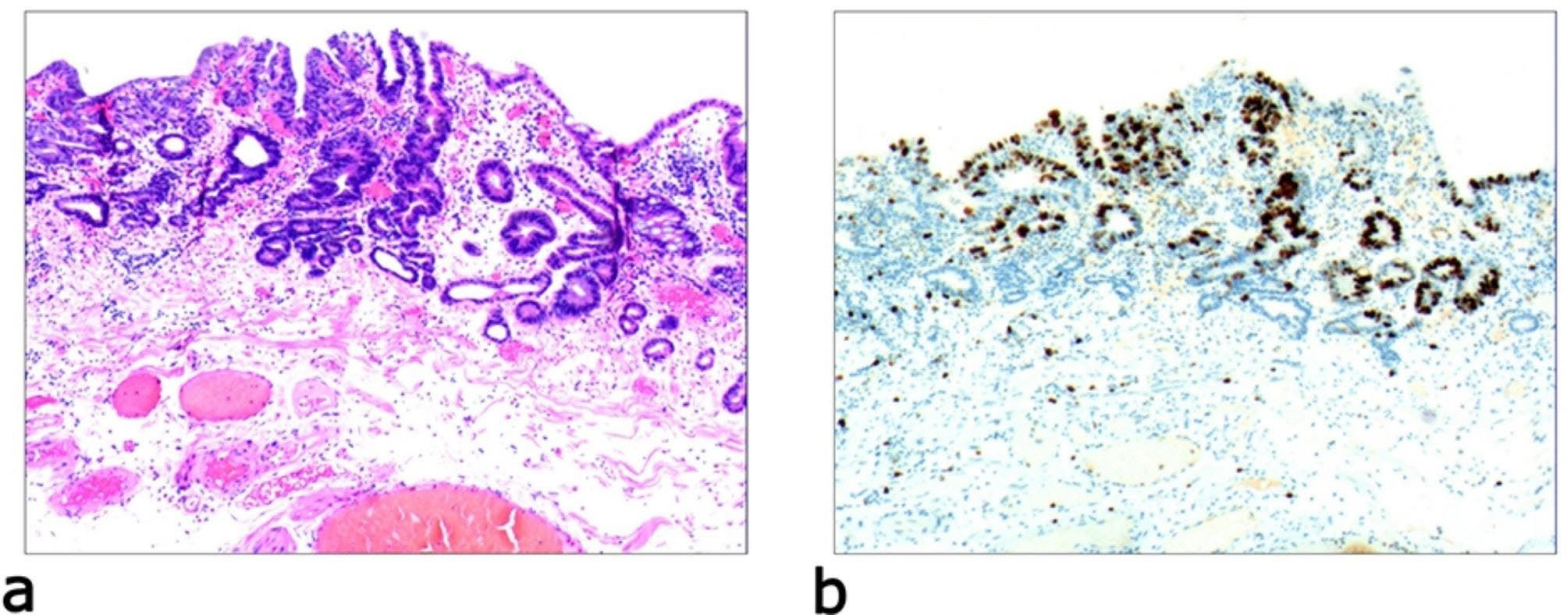
Glandular atrophy low-grade intraepithelial neoplasia, **a**: the epithelial cells present with mild to moderate heteromorphosis. The nucleus is elongated but still polar, located at the base of the glandular epithelium. The low-grade intraepithelial neoplasia occupies part of or full-thickness of the gastric mucosa. H&E staining (× 40). **b**: the ki-67-positive cells account for 30–40%. EnVision method (× 40)

**Fig. 6:**
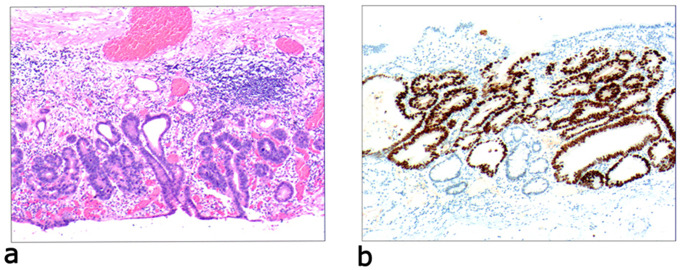
Glandular atrophy high-grade intraepithelial neoplasia, **a**: the epithelial cells present with obvious heteromorphosis. The shape of cells changes from columnar to cubic, with large nucleus, increased ratio of nucleus to cytoplasm, obvious nucleoli, and increased mitotic figures. High-grade intraepithelial neoplasia occupies almost the full-thickness of the gastric mucosa. H&E staining (× 40). **b**: positive expression of p53 protein. EnVision method (× 40)

**Fig. 7:**
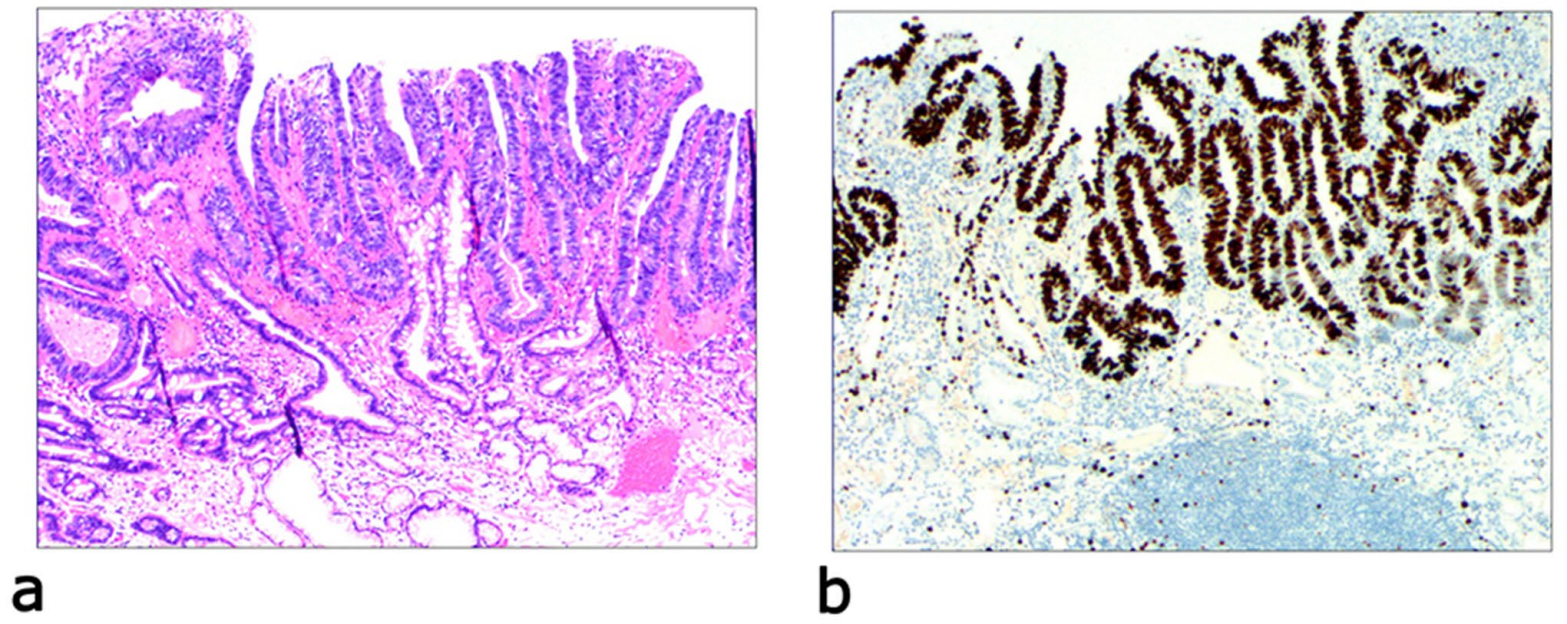
Intramucosal gastric adenocarcinoma, **a**: the cancerous tissue forms a glandular papillary structure, occupying nearly the entire layer of the gastric mucosa. H&E staining (× 40). **b**: the ki-67-positive cells account for 70–80%. EnVision method (× 40)

Due to differences in the etiology and duration of gastric mucosal atrophy, the degree of gastric mucosal damage varies, and the pathological morphology is different. Simple mild glandular atrophy of lamina propria occurred initially at the proliferation area of the deep gastric pits, and the isthmus and neck of the gastric glands, which presented with roughly normal structure in the morphology, reduced number of mitotic figures, and ki67 proliferation index in the proliferation area (Fig. 2). Symptoms in patients at this stage can manifest as degenerative changes in the elderly. Simple severe glandular atrophy of lamina propria presented with more markedly reduced number of mitotic figures and ki67 proliferation index in the proliferation area of the deep gastric pit, and the isthmus and neck of the gastric glands (Fig. 3). Glandular atrophy proliferation and transformation refers to formation of single ducts or multiple ducts, or patchy hyperplasia in the proliferation areas of the deep gastric pits, and the isthmus and neck of the gastric glands. The hyperplastic glands were slightly expanded, and the number of mitotic figures and ki67 proliferation index in the proliferation area were both increased (Fig. 4). Glandular atrophy low-grade intraepithelial neoplasia presented with mild to moderate heteromorphosis in the proliferation areas or proliferation band areas of the deep gastric pit, and the isthmus and neck of the gastric glands. Nuclei became elongated but were still polar and located at the base of the glandular epithelium. Small to medium-sized nucleoli were visible in the nucleus (Fig. 5). Glandular atrophy high-grade intraepithelial neoplasia presented with obvious heteromorphosis in the proliferation areas or proliferation band areas of the deep gastric pit, and the isthmus and neck of the gastric glands. The shape of cells changed from columnar to cubic, with large nucleus, increased ratio of nucleus to cytoplasm, obvious nucleoli, and increased mitotic figures. High-grade intraepithelial neoplasia occupied almost the full-thickness of the gastric mucosa (Fig. 6). In gastric adenocarcinoma, the cancerous tissue formed a glandular papillary structure, occupying the entire layer of the gastric mucosa. Cytologically, enlarged nucleoli and increased ratio of nucleus to cytoplasm were visible. Enlarged and obvious nucleoli could be seen in ≥ 50% nuclei (Fig. 7). The diagnostic criteria are shown in Table 2.

**Table 2:** Clinicopathological features of glandular atrophy of lamina propria in 896 cases

Type	Histopathological features (deep area of the gastric fovea, the isthmus of the gastric gland and the proliferating area of the glandular neck)				Immunophenotype	Clinically relevant
	Structure	Morphology	Mitotic pattern	Cell nucleus		
Lamellar gland simple atrophy	Generally normal Glands number ↓ Gland volume ↓ Glandular nterval ↑	No significant change The number of basal glands of the gastric gland is reduced by 1/3 of the original glands	0 ~ 2 /10HPF	Generally normal	MUC6 positive cells ↓; MUC5AC positive cells ↓ (possible). The positive number of ki67 in the proliferating zone 5% ~ 15%	Mainly occur in the elderly; Caused by the slow down of normal physiological migration of the proliferative zone.
Lamina propria simple heavy atrophy	Generally normal Glands number ↓ Gland volume ↓ Glandular nterval ↑↑ Collagen fibers ↑ Lymphocytes ↑ Plasma cells ↑ Smooth muscle fiber bundles (+)	No significant change The number of basal glands of the gastric gland is reduced by more than 1/3 of the original glands.	0 ~ 3 /10HPF	Generally normal	MUC6 positive cells ↓↓ MUC5AC positive cells ↓. The positive number of ki67 in the proliferating zone 2% ~ 10%	Degenerative changes in the elderly; Can be seen in the influence of chemical stimulation, autoimmune diseases and genetic factors.
Glandular atrophy, proliferation and transformation	Cells proliferated significantly	The adenomatous proliferation: single to multi-glandular tubules; Lumpy distribution; Gradually enlargement	1 ~ 3 /HPF	Volume increased (1 ~ 2 times of normal nucleus) nucleoli: small	CK20 (+/-) CK7 (+/-) The positive number of ki67 20–30%	Atrophy of the pyloric/fundus/cardia glands; Mainly seen in gastric mucosa affected by H.pylori infection and other factors.
Glandular atrophy low grade intraepithelial neoplasia	Atypical epithelial cell hyperplasia: light to moderate	Low grade internal neoplasia occupies part or whole layer of gastric mucosa	2 ~ 4 /HPF	The nuclei: elongated and polar; The nucleoli: small to medium size	CDX2 (+/-) Villin (+/-) CK20 (+/-). The positive number of ki67 30–40%.	Requires follow-up or endoscopic treatment
Glandular atrophy high-grade intraepithelial neoplasia	Atypical epithelial cell hyperplasia: medium to severe	High-grade internal neoplasia occupy part or all of the gastric mucosa; Cells changed from columnar to cube-shaped	2 ~ 6 /HPF	The nuclei: large; The nucleolar ratio: increased; The nucleoli: obvious.	CDX2 (+) villin (+) p53 (+) The number of ki67 positive 30–50%	Requires endoscopic ESD resection
Gastric intramucosal carcinoma	Evolve into cancer cells	Cancer cells confined to the mucosa, part of the mucosa or the whole mucosa; Do not invade the submucos.		The nuclei: large or irregularity; The nucleoli: obvious or irregularity.	CDX2 (+) villin (+) p53 (+) The number of ki67 positive 30–80%	Histopathological papillary, glandular, sig-ring cells and undifferentiated carcinoma

### Immunohistochemical staining results

MUC6 was positively expressed in the normal gastric mucosa, fundic gland, and pyloric gland, and the number of MUC6-positive cells is one of the indicators to measure the degree of glandular atrophy of lamina propria. MUC5AC was positively expressed in surface epithelial cells (gastric pits) and mucous neck cells of the normal gastric mucosa, and the number of MUC5AC-positive cells could be used to determine whether there is atrophy of surface epithelial cells and mucous neck cells, and to determine whether there is a proliferation disorder, that is, whether surface epithelial cells and mucous neck cells have migrated down into the lamina propria. CK7 and CK20 are often negatively expressed in the normal gastric mucosa and positively expressed in the proliferation and transformation stages of glandular atrophy. The expressions of villin and CDX2 were negative in the normal gastric mucosa, while various degrees of villin and CDX2 positive expressions were observed in the intraepithelial neoplasia period of glandular atrophy. p53 and Ki-67 are important indicators to determine the cancerization of mucosal atrophy. The results of immunohistochemical staining are shown in Table 2.

## Discussion

Inflammation of the gastric mucosa can induce mucosal atrophy, which involves the disappearance and metaplasia of normal glands [17, 18]. The incidence of chronic atrophic gastritis remains unclear. The disease is often not diagnosed at an early stage but in the advanced stage [19]. Chronic atrophic gastritis is considered as a precursory lesion of gastric cancer, and the common causes of this precancerous condition are *H.pylori* infection and autoimmune gastritis, which lead to low gastric acid, hypergastrinemia, and inadequate secretion of intrinsic factor [20, 21]. What’s more, Autoimmune gastritis with Helicobacter pylori infection is a high risk factor for precancerous lesions, while autoimmune gastritis in the absence of H. pylori infection does not represent precancerous lesions [22–25]. In terms of histopathology, chronic atrophic gastritis is often accompanied by atrophy and inflammation, which is often subsumed under atrophic gastropathy in actual pathological work [26, 27]. In this study, we propose the term gastric mucosal atrophic lesion for this pathology.

The description of normal histology of gastric mucosa is as follows. The bottom of each gastric pit is connected to 3–5 glands called gastric units, which are the basic structural unit of the gastric mucosa. The gastric unit is composed of gastric pits and gastric glands. The gastric gland is further divided into the isthmus, glandular neck, and base. The deep area of the gastric pits, the isthmus of the gastric gland, and the upper area of glandular neck together are called the proliferation area or proliferation band. The cells from the top of the fundic gland to the deep gastric pits are small in volume and have short columnar form. These cells are mainly proliferating stem cells. Some of them migrate upwards and differentiate into surface mucus cells, while some stay local or migrate downwards, differentiating into other fundic glandular cells [28–31]. Histologically, however, there are differences in the pylorus, body-base, and cardiac areas. These differences, also taken into consideration in Sidney’s classification scheme, make it difficult to evaluate the disappearance of the glandular elements in the different gastric districts. The structure and function of the glandular layer, in fact, vary depending on the different areas of the stomach. The foveolae are larger in the areas of the cardial and pyloric mucosa than elsewhere. The foveolae occupy approximately half the thickness of the mucosa in the cardial and pyloric areas. The foveolae in the mucosa of the oxyntic gland constitute only about one-fifth of the thickness of the mucosa. The glands are straight rather than coiled and are tightly packed relative to the cardial and pyloric mucosa [32]. In this study, we observed 896 cases of glandular atrophy of gastric mucosa and found that when the gastric mucosal epithelium was affected by infection, chemical stimuli, immune factors, and genetic factors, stem cells in the gastric mucosal proliferation area could not differentiate and migrate normally, and gastric mucosal atrophy then occurred, which is termed as gastric mucosal atrophic lesion [16]. With respect to the formation of gastric mucosal atrophy—on the one hand, due to the large proliferation of cells in the proliferating area, the downward migration function was insufficient in the normal physiological process, resulting in the atrophy of the pyloric gland/fundus gastric gland/cardiac gland, which was morphologically manifested as atrophy of lamina propria, which is morphologically manifested as glandular atrophy of the lamina propria; on the other hand, excessive proliferation in the proliferation area results in the transformation of downward migrating cells, which is morphologically manifested as intraepithelial neoplasia. This is termed as gastric mucosal tumorigenesis caused by glandular atrophy of the gastric mucosa. We also found that not only the degree of gland loss, but also more importantly, the degree of gland proliferation, should be distinguished histologically in gastric mucosal atrophy. Therefore, we propose classifying the degree of gland loss in the lamina propria of gastric mucosa as mild glandular atrophy of the lamina propria and severe glandular atrophy of the lamina propria. Additionally, we propose categorizing the degree of proliferation in the lamina propria of gastric mucosa into proliferation and transformation, or intraepithelial neoplasia. We devised the following histopathological grades based on the occurrence and development process of glandular atrophy of gastric mucosa, and the tissue morphological characteristics from benign to malignant—simple mild glandular atrophy of lamina propria, simple severe glandular atrophy of lamina propria, proliferation and transformation of glandular atrophy, low-grade intraepithelial of glandular atrophy, and high-grade intraepithelial of glandular atrophy. This grading is mainly proposed based on changes in the histological structure and cell morphology of the gastric mucosa, as well as the pattern of the occurrence and development of lesions. Including the histopathological grading of glandular atrophy of lamina propria of gastric mucosa into the clinicopathological report can guide clinicians in precise treatment and tracking of malignant cell transformation, which is very important for controlling the occurrence and development of gastric cancer.

Gastric cancer remains one of the most common causes of death worldwide, and this is mainly because most patients with gastric cancer are diagnosed in the middle to advanced stages. The survival rates of such patients are very low even with perioperative or adjuvant chemotherapy [33–36]. In recent years, it has been reported that screening and diagnosis of early gastric cancer can be achieved by examining peripheral blood, urine, saliva, gastric juice, and specific biomarkers [37, 38]. It has also been reported that with the use of Linked Color Imaging, early gastric cancer can be diagnosed based on its specific color [39]. The current study found that the precancerous lesions before tumor formation, especially in the proliferation and transformation period of glandular atrophy of lamina propria, should be mentioned in the pathological report of the gastric mucosal biopsy specimen, and this can help clinicians to carry out effective control measures. We also found that in the early stage of glandular atrophy of lamina propria, both, the number of mitotic figures and the ki67 proliferation index decreased in the proliferation area of the deep gastric pits, and the isthmus and neck of the gastric glands. Subsequently, the number of mitotic figures and the ki67 proliferation index both increased in the proliferation area. Finally, this led to the occurrence of gastric adenocarcinoma. This study highlights the glandular atrophy proliferation and transformation period in glandular atrophy of gastric mucosa, during which cells overproliferated, and the number of mitotic figures and the ki67 proliferation index increased, suggesting the need for close biopsy review. Our study is of great significance for the histopathological characteristics of glandular atrophy of the lamina propria of gastric mucosa, the histopathological diagnostic criteria and immunophenotypic characteristics leading to the early and precancerous stages of gastric adenocarcinoma, as well as the study of the occurrence and development of gastric mucosal atrophy.

CDX2 is a gut-specific transcription factor that plays an important role in regulating intestinal epithelial development, differentiation, phenotypic maintenance, and function [40]. In adults, CDX2 expression is strictly restricted to the intestine and is not expressed in normal gastric mucosa [41]. Ectopic expression of CDX2 is closely related to IM. The results of transgenic mice confirmed that CDX2 is sufficient to cause a wide range of IM in normal gastric mucosa, which not only has morphological transformation, but also has partial intestinal functions, confirming that CDX2 can directly regulate the transcription of many intestinal specific molecules and induce IM. Therefore, ectopic expression and activation of CDX2 are key events in IM occurrence [42, 43]. The results of this study indicated that villus proteins and CDX2 were positively expressed in different degrees during adenoatrophic intraepithelial neoplasia, which was consistent with previous research results.

## Conclusion

In summary, in this study we have proposed that gastric mucosal atrophy originates from the proliferation area, and we have presented the histopathological diagnostic criteria for glandular atrophy of lamina propria of gastric mucosa leading to early gastric adenocarcinoma and precancerous stage, which can be helpful for clinicians to offer accurate treatment and track malignant cell transformation. This is of great significance in controlling the occurrence and development of gastric cancer. The proliferation area of the gastric mucosa contains a variety of poorly differentiated cells with diverse differentiation directions. It is unclear as to which kind of cells in the proliferation area that the gastric mucosa atrophy originates in, and this requires further observation in a large number of cases as well as research studies on molecular pathology.

## Data Availability

All data generated or analysed during this study are included in this article. Further enquiries can be directed to the corresponding author.
